# Supplementary material for: Using continuous directed evolution to improve enzymes for plant applications
Source: Plant Physiol. 2021 Oct 27;188(2):971–83. doi: 10.1093/plphys/kiab500 (PMC8825276; doi:10.1093/plphys/kiab500)
Supplement: kiab500_Supplementary_Data [file kiab500_supplementary_data.pdf]

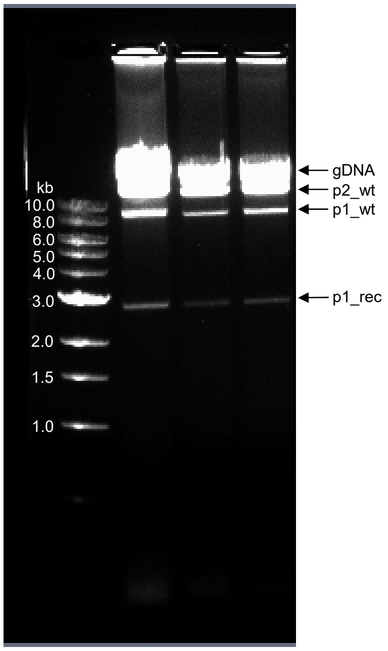

Supplemental Figure S1. Agarose gel of total DNA extracted from individual colonies of GA-Y319 transformed with the ScaI-digested GR-306MP plasmid. p2_wt is ~13.5 kb, p1_wt is ~8.9 kb and p1 recombined with *MhTHI4* (p1_rec) is ~2.8 kb, the predicted size for *MhTHI4* (2.1 kb p1 backbone + 0.7 kb *MhTHI4*). Colonies whose p1_rec bands are of incorrect size or that have additional plasmid bands are discarded. gDNA = genomic DNA.

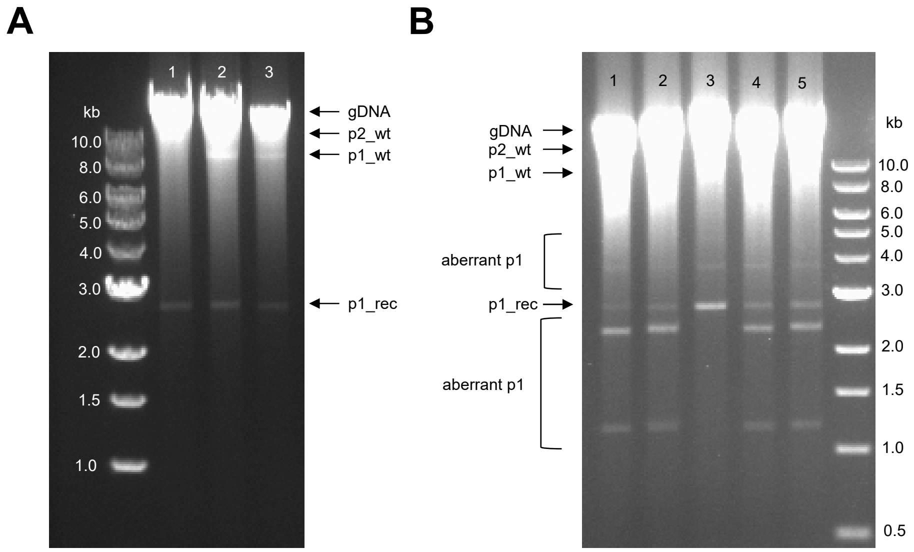

Supplemental Figure S2. Expected agarose gel band patterning of total DNA extracts of BY4741 *thi4*Δ cells harboring *MhTHI4*. A, DNA from three individual isolates immediately following protoplast fusion. p2_wt and p1_rec will be present and p1_wt is often also present (lanes 2 and 3) but can be absent (lane 1). B, DNA from five individual isolates after multiple passages under selection. The image captured is overexposed to show minor bands. p2_wt and p1_rec will be present; p1_wt will likely be lost over multiple passages (p1_wt and p2_wt are obscured due to overexposure). Extra bands (attributable to aberrant forms of p1 because they disappear if protease treatment is omitted from the DNA isolation protocol) commonly appear after multiple passages and may also sometimes appear following protoplast fusion. p2_wt is ~13.5 kb, p1_wt is ~8.9 kb, and p1_rec is ~2.8 kb (2.1 kb p1 backbone + 0.7 kb *MhTHI4*). gDNA = genomic DNA.

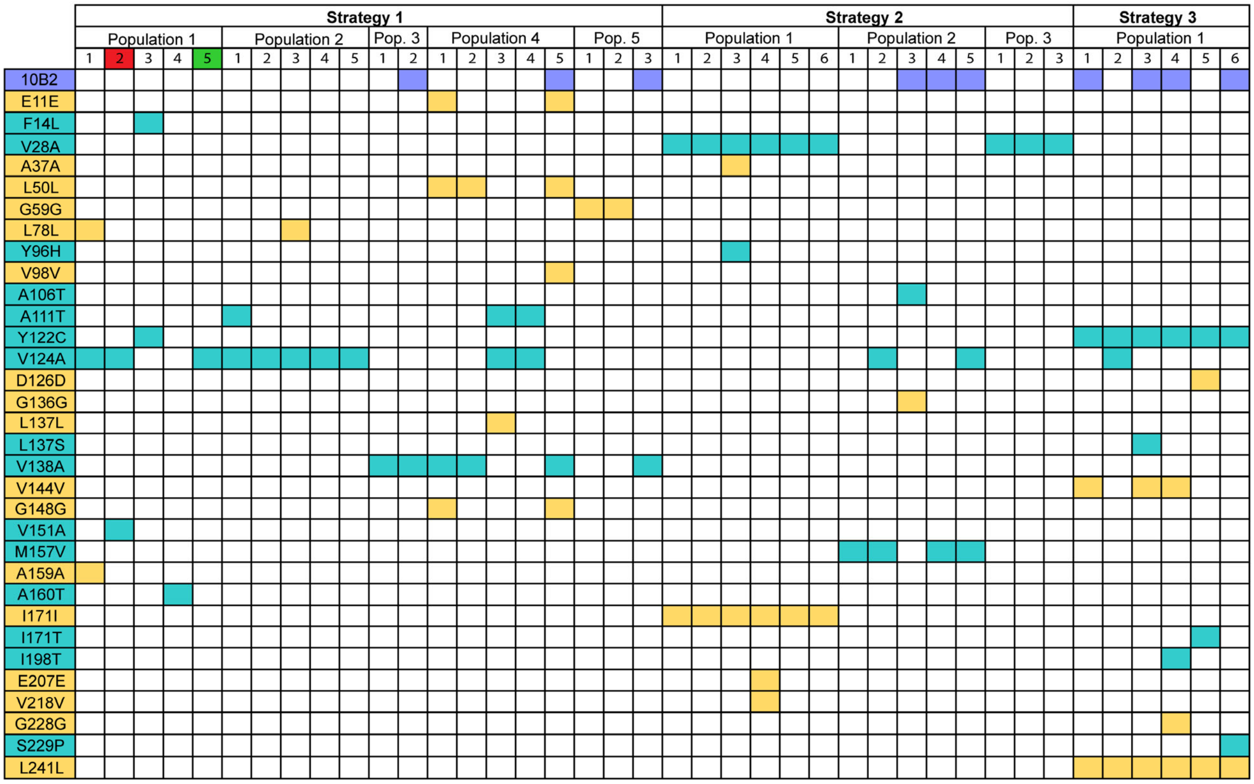

Supplemental Figure S3. Mutations found in the promoter and coding regions of *MhTHI4*. Promoter mutations are highlighted in violet, synonymous mutations in the coding region are gold, and non-synonymous mutations are turquoise. In the header, the V124A and V151A mutations, which have respectively positive and negative effects, are highlighted in green and red.

Supplemental Table S1. Sequences of vectors and genes used in this study.

Name	Sequence (5′–3′)	Purpose
ScTHI4	ATGTCTGCTACCTCTACTGCTACTTCCACAAGTGCCTCTCAATTGCACTTAAACTCTACTCCAGTTACTCACT<br>GCTTATCTGACATCGTTAAGAAAGAAGATTGGTCTGACTTTAAATTTGCTCCCATCCGCGAATCCACTGTCTC<br>TCGTGCTATGACTTCTCGTTATTTCAAGGATCTTGACAAGTTTGCCGTTTCTGACGTGATTATTGTCGGTGCG<br>GGCTCTTCAGGTTTATCCGCCGCTTACGTCATCGCCAAGAACAGACCAGACTTGAAGGTTTGTATTATCGAAA<br>GTTCAGTTGCACCAGGTGGTGGTAGTTGGTTGGGTGGTCAATTATTTAGTGCCATGGTTATGAGAAAACCAGC<br>TCATTTGTTCTTACAAGAGTTGGAAATCCCTTACGAAGACGAAGGTGACTATGTTGTCGTTAAACATGCCGCT<br>TTGTTCATCTCTACTGTCCTTTCAAAGGTCTTGCAATTACCAAATGTTAAACTGTTCAATGCTACCTGTGTTG<br>AAGATTTGGTTACCAGACCACCTACCGAAAAGGGCGAAGTCACCGTTGCTGGTGTTGTCACCAACTGGACGTT<br>AGTTACCCAAGCTCACGGTACTCAATGTTGCATGGACCCTAACGTAATTGAATTGGCAGGTTACAAAAATGAC<br>GGAACTCGTGACTTGAGTCAAAAGCATGGTGTCATTTTATCCACTACCGGTCATGATGGTCCATTTGGTGCTT<br>TCTGCGCCAAGAGAATCGTCGACATTGATCAAAACCAAAAATTGGGCGGTATGAAGGGTCTGGACATGAACCA<br>TGCCGAACACGATGTCGTTATTCACTCTGGTGCATACGCCGGTGTTGACAACATGTACTTTGCTGGTATGGAA<br>GTTGCTGAACTGGATGGATTAAACCGTATGGGTCCAACTTTTGGAGCTATGGCTTTGAGTGGTGTTCATGCTG<br>CTGAGCAAATTTTGAAACACTTTGCTGCTTAG	Positive control for complementation assay and continuous directed evolution (OrthoRep)
MhTHI4	ATGGAAAAGATTGTTTCTGCTGGTATTGTTGAATCATACTTCGATAAGTTGAGAAGAAATTTGGTTTTAGATG<br>TTGCAATCGTTGGTGGTGGTCCATCTGGTTTAGTTGCTGCATATTACTTGGCTAAAGCAGGTAGAAGAGTTGC<br>TTTGTTCGAAAGAAAGTTGGCTCCAGGTGGTGGTATGTGGGGTGGTGCTATGATGTTCAACGATATCGTTGTT<br>CAATCTGATGCTTTGCCAATCTTGGAAGAATTGGGTGTTTCATACAGACATTACAGAGGTGACGCTTATTTGG<br>TTGATTCTGTTCATGCAACTGCTGCATTGATCTATGCTGCAACTAGAGCTGGTGCAACAATTTTCAATTGTTA<br>CTCAGTTGAAGATGTTGTTTTTAAAGATGAAAGAGTTGCTGGTTTAGTTGTTAATTGGGCACCAGTTATTAGA<br>GAGGGTATGCACGTTGATCCATTAGTTATTATGGCTACTGCAGTTTTGGAAGGTACAGGTCACGATTGTGCTA<br>TTGCAAGATTAGTTGCTAGAAAGAATGGTGTTAGATTGAATACTCCAACAGGTGAAGTTATTGGTGAAAGATC<br>TTTGTCAATCGAAGAAGCTGAAAGAACTACAGTTGAAAACACAAAGGAAATATATCCAGGTTTGTTTGTTTCT<br>GGTATGGCTGCAAATGGTGTTTCTGGTTCTTTTAGAATGGGTCCAATTTTTGGTGGTATGTTGTTATCTGGTA<br>AAAAGGCTGCACAAATGATTTGTGATTCATTGTAA	Complementation assay and continuous directed evolution (OrthoRep)
ArEc-TDH3	GAGCTCCAGTTCGAGTTTATCATTATCAATACTGCCATTTCAAAGAATACGTAAATAATTAATAGTAGTGATT<br>TTCCTAACTTTATTTAGTCAAAAAATTAGCCTTTTAATTCTGCTGTAACCCGTACATGCCCAAAATAGGGGGC<br>GGGTTACACAGAATATATAACATCGTAGGTGTCTGGGTGAACAGTTTATTCCTGGCATCCACTAAATATAATG<br>GAGCCCGCTTTTTAAGCTGGCATCCAGAAAAAAAAAGAATCCCAGCACCAAAATATTGTTTTCTTCACCAACC<br>ATCAGTTCATAGGTCCATTCTCTTAGCGCAACTACAGAGAACAGGGGCACAAACAGGCAAAAAACGGGCACAA<br>CCTCAATGGAGTGATGCAACCTGCCTGGAGTAAATGATGACACAAGGCAATTGACCCACGCATGTATCTATCT<br>CATTTTCTTACACCTTCTATTACCTTCTGCTCTCTCTGATTTGGAAAAAGCTGAAAAAAAAGGTTGAAACCAG<br>TTCCCTGAAATTATTCCCCTACTTGACTAATAAGTATATAAAGACGGTAGGTATTGATTGTAATTCTGTAAAT<br>CTATTTCTTAAACTTCTTAAATTCTACTTTTATAGTTAGTCTTTTTTTTAGTTTTAAAACACCAAGAACTTAG<br>TTTCGAATAAACACACATAAACAAACAAAGAATTCTACGCGTAGCATGCCTCGAGGCGAATTTCTTATGATTT<br>ATGATTTTTATTATTAAATAAGTTATAAAAAAAATAAGTGTATACAAATTTTAAAGTGACTCTTAGGTTTTAA<br>AACGAAAATTCTTATTCTTGAGTAACTCTTTCCTGTAGGTCAGGTTGCTTTCTCAGGTATAGCATGAGGTCGC<br>TCACTAGTCCATGGACGCGTGGTACCGGCCGCGTTGCTGGCGTTTTTCCATAGGCTCCGCCCCCCTGACGAGC<br>ATCACAAAAATCGACGCTCAAGTCAGAGGTGGCGAAACCCGACAGGACTATAAAGATACCAGGCGTTTCCCCC<br>TGGAAGCTCCCTCGTGCGCTCTCCTGTTCCGACCCTGCCGCTTACCGGATACCTGTCCGCCTTTCTCCCTTCG<br>GGAAGCGTGGCGCTTTCTCATAGCTCACGCTGTAGGTATCTCAGTTCGGTGTAGGTCGTTCGCTCCAAGCTGG<br>GCTGTGTGCACGAACCCCCCGTTCAGCCCGACCGCTGCGCCTTATCCGGTAACTATCGTCTTGAGTCCAACCC<br>GGTAAGACACGACTTATCGCCACTGGCAGCAGCCACTGGTAACAGGATTAGCAGAGCGAGGTATGTAGGCGGT<br>GCTACAGAGTTCTTGAAGTGGTGGCCTAACTACGGCTACACTAGAAGAACAGTATTTGGTATCTGCGCTCTGC<br>TGAAGCCAGTTACCTTCGGAAAAAGAGTTGGTAGCTCTTGATCCGGCAAACAAACCACCGCTGGTAGCGGTGG<br>TTTTTTTGTTTGCAAGCAGCAGATTACGCGCAGAAAAAAAGGATCTCAAGAAGATCCTTTGATCTTTTCTACG<br>GGGTCTGACGCTCAGTGGAACGAAAACTCACGTTAAGGGATTTTGGTCATGACTGATCCTTCAACTCAGCAAA<br>AGTTCGATTTATTCAACAAAGCCACGTTGTGTCTCAAAATCTCTGATGTTACATTGCACAAGATAAAAATATA<br>TCATCATGAACAATAAAACTGTCTGCTTACATAAACAGTAATACAAGGGGTGTTATGAGCCATATTCAACGGG<br>AAACGTCTTGCTCCAGGCCGCGATTAAATTCCAACATGGATGCTGATTTATATGGGTATAAATGGGCTCGCGA<br>TAATGTCGGGCAATCAGGTGCGACAATCTATCGATTGTATGGGAAGCCCGATGCGCCAGAGTTGTTTCTGAAA<br>CATGGCAAAGGTAGCGTTGCCAATGATGTTACAGATGAGATGGTCAGACTAAACTGGCTGACGGAATTTATGC<br>CTCTTCCGACCATCAAGCATTTTATCCGTACTCCTGATGATGCATGGTTACTCACCACTGCGATCCCCGGGAA<br>AACAGCATTCCAGGTATTAGAAGAATATCCTGATTCAGGTGAAAATATTGTTGATGCGCTGGCAGTGTTCCTG<br>CGCCGGTTGCATTCGATTCCTGTTTGTAATTGTCCTTTTAACAGCGATCGCGTATTTCGTCTCGCTCAGGCGC<br>AATCACGAATGAATAACGGTTTGGTTGATGCGAGTGATTTTGATGACGAGCGTAATGGCTGGCCTGTTGAACA<br>AGTCTGGAAAGAAATGCATAAGCTTTTGCCATTCTCACCGGATTCAGTCGTCACTCATGGTGATTTCTCACTT<br>GATAACCTTATTTTTGACGAGGGGAAATTAATAGGTTGTATTGATGTTGGACGAGTCGGAATCGCAGACCGAT<br>ACCAGGATCTTGCCATCCTATGGAACTGCCTCGGTGAGTTTTCTCCTTCATTACAGAAACGGCTTTTTCAAAA<br>ATATGGTATTGATAATCCTGATATGAATAAATTGCAGTTTCATTTGATGCTCGATGAGTTTTTCTAATCAGAA<br>TTGGTTAATTGGTTGTAACACGGTCCTTTTCATCACGTGCTATAAAAATAATTATAATTTAAATTTTTTAATA<br>TAAATATATAAATTAAAAATAGAAAGTAAAAAAAGAAATTAAAGAAAAAATAGTTTTTGTTTTCCGAAGATGT<br>AAAAGACTCTAGGGGGATCGCCAACAAATACTACCTTTTATCTTGCTCTTCCTGCTCTCAGGTATTAATGCCG<br>AATTGTTTCATCTTGTCTGTGTAGAAGACCACACACGAAAATCCTGTGATTTTACATTTTACTTATCGTTAAT<br>CGAATGTATATCTATTTAATCTGCTTTTCTTGTCTAATAAATATATATGTAAAGTACGCTTTTTGTTGAAATT<br>TTTTAAACCTTTGTTTATTTTTTTTTCTTCATTCCGTAACTCTTCTACCTTCTTTATTTACTTTCTAAAATCC<br>AAATACAAAACATAAAAATAAATAAACACAGAGTAAATTCCCAAATTATTCCATCATTAAAAGATACGAGGCG<br>CGTGTAAGTTACAGGCAAGCGATCCGTCCTAAGAAACCATTATTATCATGACATTAACCTATAAAAATAGGCG<br>TATCACGAGGCCCTTTCGTCTCGCGCGTTTCGGTGATGACGGTGAAAACCTCTGACACATGCAGCTCCCGGAG<br>ACGGTCACAGCTTGTCTGTAAGCGGATGCCGGGAGCAGACAAGCCCGTCAGGGCGCGTCAGCGGGTGTTGGCG<br>GGTGTCGGGGCTGGCTTAACTATGCGGCATCAGAGCAGATTGTACTGAGAGTGCACCACGCTTTTCAATTCAA<br>TTCATCATTTTTTTTTTATTCTTTTTTTTGATTTTTTAAGAGCTTGGTGAGCGCTAGGAGTCACTGCCAGGTA<br>TCGTTTGAACACGGCATTAGTCAGGGAAGTCATAACACAGTCCTTTCCCGCAATTTTCTTTTTCTATTACTCT<br>TGGCCTCCTCTAGTACACTCTATATTTTTTTATGCCTCGGTAATGATTTTCATTTTTTTTTTTCCACCTAGCG<br>GATGACTCTTTTTTTTTCTTAGCGATTGGCATTATCACATAATGAATTATACATTATATAAAGTAATGTGATT<br>TCTTCGAAGAATATACTAAAAAATGAGCAGGCAAGATAAACGAAGGCAAAGATGACAGAGCAGAAAGCCCTAG<br>TAAAGCGTATTACAAATGAAACCAAGATTCAGATTGCGATCTCTTTAAAGGGTGGTCCCCTAGCGATAGAGCA<br>CTCGATCTTCCCAGAAAAAGAGGCAGAAGCAGTAGCAGAACAGGCCACACAATCGCAAGTGATTAACGTCCAC<br>ACAGGTATAGGGTTTCTGGACCATATGATACATGCTCTGGCCAAGCATTCCGGCTGGTCGCTAATCGTTGAGT<br>GCATTGGTGACTTACACATAGACGACCATCACACCACTGAAGACTGCGGGATTGCTCTCGGTCAAGCTTTTAA<br>AGAGGCCCTAGGGGCCGTGCGTGGAGTAAAAAGGTTTGGATCAGGATTTGCGCCTTTGGATGAGGCACTTTCC	Nuclear plasmid: negative control for complementation assay and expression of error-prone polymerase in continuous directed evolution (OrthoRep)

	AGAGCGGTGGTTGATCTTTCGAACAGGCCGTACGCAGTTGTCGAACTTGGTTTGCAAAGGGAGAAAGTAGGTG<br>ATCTCTCTTGCGAGATGATCCCGCATTTTCTTGAAAGCTTTGCAGAGGCTAGCAGAATTACCCTCCACGTTGA<br>TTGTCTGCGAGGCAAGAATGATCATCACCGTAGTGAGAGTGCGTTCAAGGCTCTTGCGGTTGCCATAAGAGAA<br>GCCACCTCGCCCAATGGTACCAACGATGTTCCCTCCACCAAAGGTGTTCTTATGTAGTGACACCGATTATTTA<br>AAGCTGCTGCATACGATATATATACATGTGTATATATGTATACCTATGAATGTCAGTAAGTATGTATACGAAC<br>AGTATGATACTGAAGATGACAAGGTAATGCATCATTCTATACGTGTCATTCTGAACGAGGCGCGCTTTCCTTT<br>TTTCTTTTTGCTTTTTCTTTTTTTTTCTCTTGAACTCGACGGATCATATATGCGGTGTGAAATACCGCACAGA<br>TGCGTAAGGAGAAAATACCGCATCAGG	
GR-306MP	GAAGATCCTTTGATCTTTTCTACGGGGTCTGACGCTCAGTGGAACGAAAACTCACGTTAAGGGATTTTGGTCA<br>TGAGATTATCAAAAAGGATCTTCACCTAGATCCTTTTAAATTAAAAATGAAGTTTTAAATCAATCTAAAGTAT<br>ATATGAGTAAACTTGGTCTGACAGTTACCAATGCTTAATCAGTGAGGCACCTATCTCAGCGATCTGTCTATTT<br>CGTTCATCCATAGTTGCCTGACTCCCCGTCGTGTAGATAACTACGATACGGGAGGGCTTACCATCTGGCCCCA<br>GTGCTGCAATGATACCGCGAGACCCACGCTCACCGGCTCCAGATTTATCAGCAATAAACCAGCCAGCCGGAAG<br>GGCCGAGCGCAGAAGTGGTCCTGCAACTTTATCCGCCTCCATCCAGTCTATTAATTGTTGCCGGGAAGCTAGA<br>GTAAGTAGTTCGCCAGTTAATAGTTTGCGCAACGTTGTTGCCATTGCTACAGGCATCGTGGTGTCACGCTCGT<br>CGTTTGGTATGGCTTCATTCAGCTCCGGTTCCCAACGATCAAGGCGAGTTACATGATCCCCCATGTTGTGCAA<br>AAAAGCGGTTAGCTCCTTCGGTCCTCCGATCGTTGTCAGAAGTAAGTTGGCCGCAGTGTTATCACTCATGGTT<br>ATGGCAGCACTGCATAATTCTCTTACTGTCATGCCATCCGTAAGATGCTTTTCTGTGACTGGTGAGTACTCAA<br>CCAAGTCATTCTGAGAATAGTGTATGCGGCGACCGAGTTGCTCTTGCCCGGCGTCAATACGGGATAATACCGC<br>GCCACATAGCAGAACTTTAAAAGTGCTCATCATTGGAAAACGTTCTTCGGGGCGAAAACTCTCAAGGATCTTA<br>CCGCTGTTGAGATCCAGTTCGATGTAACCCACTCGTGCACCCAACTGATCTTCAGCATCTTTTACTTTCACCA<br>GCGTTTCTGGGTGAGCAAAAACAGGAAGGCAAAATGCCGCAAAAAAGGGAATAAGGGCGACACGGAAATGTTG<br>AATACTCATACTCTTCCTTTTTCAATATTATTGAAGCATTTATCAGGGTTATTGTCTCATGAGCGGATACATA<br>TTTGAATGTATTTAGAAAAATAAACAAATAGGGGTTCCGCGCACATTTCCCCGAAAAGTGCCACCTGACGTCT<br>AAGAAACCATTATTATCATGACATTAACCTATAAAAATAGGCGTATCACGAGGCCCTTTCGTCTCACGCGTTT<br>CGGTGATGACGGTGAAAACCTCTGACACATGCAGCTCCCGGAGACGAGTACTATAATATATGAATTACATTAT<br>TAATTTAAAAGATGACCTATACATAGGAAGATCTATAGAAACAAAAAGATTAATAACTTTCAAATATCAGAAA<br>AATGTAGAAACATGTGATAAGCTCATAGACATGTAAAATGCATGTGAGCGAGCTGATTAAGGAGAACATGCAC<br>ATGAAGCTGTACATGGAGGGCACCGTGAACAACCACCACTTCAAGTGCACATCCGAGGGCGAAGGCAAGCCCT<br>ACGAGGGCACCCAGACCATGAGAATCAAGGCGGTCGAGGGCGGCCCTCTCCCCTTCGCCTTCGACATCCTGGC<br>TACCAGCTTCATGTACGGCAGCAAAACCTTCATCAACCACACCCAGGGCATCCCCGACTTCTTTAAGCAGTCC<br>TTCCCCGAGGGCTTCACATGGGAGAGAGTCACCACATACGAAGACGGGGGCGTGCTGACCGCTACCCAGGACA<br>CCAGCCTCCAGGACGGCTGCCTCATCTACAACGTCAAGATCAGAGGGGTGAACTTCCCATCCAACGGCCCTGT<br>GATGCAGAAGAAAACACTCGGCTGGGAGGCCTCCACCGAGACCCTGTACCCCGCTGACGGCGGCCTGGAAGGC<br>AGAGCCGACATGGCCCTGAAGCTCGTGGGCGGGGGCCACCTGATCTGCAACTTGAAGACCACATACAGATCCA<br>AGAAACCCGCTAAGAACCTCAAGATGCCCGGCGTCTACTATGTGGACAGAAGACTGGAAAGAATCAAGGAGGC<br>CGACAAAGAGACCTACGTCGAGCAGCACGAGGTGGCTGTGGCCAGATACTGCGACCTCCCTAGCAAACTGGGG<br>CACAGATAAGCATGCGATGCCCCATGAAAAAAAAAAAAAAAAAAAAAAAAAAAAAAAAAAAAAAAAAAAAAA<br>AAAAAAAAAAAAAAAAAAAAAAAAAACCTGTCACCGGATGTGTTTTCCGGTCTGATGAGTCCGTGAGGACGAA<br>ACAGGGAATTGGGATGTCATGCTTTTTCGCCGGAGTCAATTAGGTCATACTTTTCTATATAATCCAAATCCCA<br>AAAATCAATTGAATGATTCTTAATATGATTTAATAGTTTATGATTATAAATGCACGTTCCTAAGAAGATCGTC<br>GTTTTGCCAGGTGACCACGTTGGTCAAGAAATCACAGCCGAAGCCATTAAGGTTCTTAAAGCTATTTCTGATG<br>TTCGTTCCAATGTCAAGTTCGATTTCGAAAATCATTTAATTGGTGGTGCTGCTATCGATGCTACAGGTGTTCC<br>ACTTCCAGATGAGGCGCTGGAAGCCTCCAAGAAGGCTGATGCCGTTTTGTTAGGTGCTGTGGGTGGTCCTAAA<br>TGGGGTACCGGTAGTGTTAGACCTGAACAAGGTTTACTAAAAATCCGTAAAGAACTTCAATTGTACGCCAACT<br>TAAGACCATGTAACTTTGCATCCGACTCTCTTTTAGACTTATCTCCAATCAAGCCACAATTTGCTAAAGGTAC<br>TGACTTCGTTGTTGTCAGAGAATTAGTGGGAGGTATTTACTTTGGTAAGAGAAAGGAAGACGATGGTGATGGT<br>GTCGCTTGGGATAGTGAACAATACACCGTTCCAGAAGTGCAAAGAATCACAAGAATGGCCGCTTTCATGGCCC<br>TACAACATGAGCCACCATTGCCTATTTGGTCCTTGGATAAAGCTAATGTTTTGGCCTCTTCAAGATTATGGAG<br>AAAAACTGTGGAGGAAACCATCAAGAACGAATTCCCTACATTGAAGGTTCAACATCAATTGATTGATTCTGCC<br>GCCATGATCCTAGTTAAGAACCCAACCCACCTAAATGGTATTATAATCACCAGCAACATGTTTGGTGATATCA<br>TCTCCGATGAAGCCTCCGTTATCCCAGGTTCCTTGGGTTTGTTGCCATCTGCGTCCTTGGCCTCTTTGCCAGA<br>CAAGAACACCGCATTTGGTTTGTACGAACCATGCCACGGTTCTGCTCCAGATTTGCCAAAGAATAAGGTCAAC<br>CCTATCGCCACTATCTTGTCTGCTGCAATGATGTTGAAATTGTCATTGAACTTGCCTGAAGAAGGTAAGGCCA<br>TTGAAGATGCAGTTAAAAAGGTTTTGGATGCAGGTATCAGAACTGGTGATTTAGGTGGTTCCAACAGTACCAC<br>CGAAGTCGGTGATGCTGTCGCCGAAGAAGTTAAGAAAATCCTTGCTTAACCTCAGACTCAGATTGGTGTTTCA<br>AAGATCCTCAAGGCAATAGAATAACAGATTTTGATAGTATTAATAAAGAATTAGGTCTTGGTAGAAGAGATGT<br>AAAATTAGATAAAGGTCATGATGATTTAATTAAATTATGTACTGAAAAAATAGATAGTACTATTAATTGCGTT<br>GCGCTCACTGCCCGCTTTCCAGTCGGGAAACCTGTCGTGCCAGCTGCATTAATGAATCGGCCAACGCGCGGGG<br>AGAGGCGGTTTGCGTATTGGGCGCTCTTCCGCTTCCTCGCTCACTGACTCGCTGCGCTCGGTCGTTCGGCTGC<br>GGCGAGCGGTATCAGCTCACTCAAAGGCGGTAATACGGTTATCCACAGAATCAGGGGATAACGCAGGAAAGAA<br>CATGTGAGCAAAAGGCCAGCAAAAGGCCAGGAACCGTAAAAAGGCCGCGTTGCTGGCGTTTTTCCATAGGCTC<br>CGCCCCCCTGACGAGCATCACAAAAATCGACGCTCAAGTCAGAGGTGGCGAAACCCGACAGGACTATAAAGAT<br>ACCAGGCGTTTCCCCCTGGAAGCTCCCTCGTGCGCTCTCCTGTTCCGACCCTGCCGCTTACCGGATACCTGTC<br>CGCCTTTCTCCCTTCGGGAAGCGTGGCGCTTTCTCATAGCTCACGCTGTAGGTATCTCAGTTCGGTGTAGGTC<br>GTTCGCTCCAAGCTGGGCTGTGTGCACGAACCCCCCGTTCAGCCCGACCGCTGCGCCTTATCCGGTAACTATC<br>GTCTTGAGTCCAACCCGGTAAGACACGACTTATCGCCACTGGCAGCAGCCACTGGTAACAGGATTAGCAGAGC<br>GAGGTATGTAGGCGGTGCTACAGAGTTCTTGAAGTGGTGGCCTAACTACGGCTACACTAGAAGAACAGTATTT<br>GGTATCTGCGCTCTGCTGAAGCCAGTTACCTTCGGAAAAAGAGTTGGTAGCTCTTGATCCGGCAAACAAACCA<br>CCGCTGGTAGCGGTGGTTTTTTTGTTTGCAAGCAGCAGATTACGCGCAGAAAAAAAGGATCTCAA	Multipurpose vector to integrate target gene into p1_wt

Supplemental Table S2. Primers used to clone and sequence *ScTHI4* and *MhTHI4*.

The overhangs that contain the restriction sites are underlined. For cloning into GR-306MP, an additional CAT (bold) after the start codon is required.

Primer name	Sequence (5′–3′)	Purpose
ScTHI4_EcoRI_F	CGATGAATTCATGTCTGCTACCTCTACTGCTACTTC	Cloning of *ScTHI4* into ArEc-TDH3
MhTHI4_EcoRI_F	CGATGAATTCATGGAAAAGATTGTTTCTGCTGG	Cloning of *MhTHI4* into ArEc-TDH3
MhTHI4_NsiI_F	CGATATG**CAT**GAAAAGATTGTTTCTGCTGGTATTG	Cloning of *MhTHI4* into GR-306MP
MhTHI4_SphI_R	ATCGGCATGCTTACAATGAATCACAAATCATTTGTGC	Cloning of *MhTHI4* into GR-306MP and ArEc-TDH3
ScTHI4_NsiI_F	CGATATG**CAT**TCTGCTACCTCTACTGCTA	Cloning of *ScTHI4* into GR-306MP
ScTHI4_SphI_R	ATCGGCATGCCTAAGCAGCAAAGTGTTTC	Cloning of *ScTHI4* into GR-306MP and ArEc-TDH3
p1_F	TTATTGGAAGATTAGTACGTCTCC	Sequencing *MhTHI4* in p1
Ribozyme_R	CCTCACGGACTCATCAGACC	Sequencing *MhTHI4* in GR-306MP and p1
MhTHI4_Int_F	GCACCAGTTATTAGAGAGGGTATG	Sequencing *MhTHI4* in GR-306MP, ArEc-TDH3 and p1
MhTHI4_Int_R	GCTTTAGCCAAGTAATATGCAGC	Sequencing *MhTHI4* in GR-306MP, ArEc-TDH3 and p1

Supplemental Table S3. Recipes of media used in OrthoRep.

Media	Components	pH	Use
Lysogeny broth (LB)	10 g/l tryptone, 5 g/l yeast extract, 10 g/l sodium chloride, 20 g/l Bacto Agar (when needed), appropriate antibiotics	7.5	Growth of ArEc-TDH3 or GR-306MP in *E. coli* TOP10
YPD	20 g/l tryptone, 10 g/l yeast extract, 2% w/v glucose, 24 g/l Bacto Agar (when needed)	--	Growth of GA-Y319, yeast transformation cell recovery
YPD + G418	20 g/l tryptone, 10 g/l yeast extract, 2% w/v glucose, 24 g/l Bacto Agar (when needed), 200 µg/ml G418	--	Growth of BY4741 *thi4*Δ
Selection medium: synthetic complete minus histidine (SC -His -Trp)	1.71 g/l yeast nitrogen base (YNB) without amino acids (AA), ammonium sulfate (AS), and thiamin, 1.4 g/l Drop-out mix synthetic minus histidine, tryptophan, uracil, and leucine (DO), 5 g/l ammonium sulfate, 20 g/l Bacto Agar (when needed), 0.076 g/l uracil, 0.38 g/l leucine, 2% w/v glucose	6.1	Growth of ArEc-TDH3, ArEc-TDH3_*ScTHI4* or ArEc-TDH3_*MhTHI4* in BY4741 *thi4*Δ
Selection medium: synthetic complete minus leucine (SC -Leu)	6.7 g/l YNB with ammonium sulfate, 1.4 g/l DO, 20 g/l Bacto Agar (when needed), 0.076 g/l uracil, 0.076 g/l tryptophan, 0.076 g/l histidine, 2% w/v glucose	6.1	Growth of GR-306MP, GR-306MP_*ScTHI4* or GR-306MP_*MhTHI4* in GA-Y319
Protoplast selection medium: synthetic complete minus histidine, leucine, tryptophan (SC -His -Leu -Trp + KCl)	1.71 g/l YNB without AA, AS, and thiamin, 1.4 g/l DO, 5 g/l ammonium sulfate, 0.6 M potassium chloride, 0.076 g/l uracil, 2% w/v glucose, 3% Bacto Agar, 300 nM thiamin	6.1	Growth of BY4741 *thi4*Δ with p2_wt, p1_wt and p1_rec during protoplast fusion
Selection medium: synthetic complete minus histidine, leucine, tryptophan plus G418 (SC -His -Leu -Trp + G418)	1.71 g/l YNB without AA, AS, and thiamin, 1.4 g/l DO, 1 g/l monosodium glutamate, 20 g/l Bacto Agar (if needed), 0.076 g/l uracil, 2% w/v glucose, plus or minus thiamin, 200 µg/ml G418	6.1	Selection of BY4741 *thi4*Δ with p2_wt, p1_wt and p1_rec
Selection medium: synthetic complete minus histidine, leucine, tryptophan (SC -His -Leu -Trp)	1.71 g/l YNB without AA, AS, and thiamin, 1.4 g/l DO, 5 g/l ammonium sulfate, 0.076 g/l uracil, 2% w/v glucose, plus or minus thiamin	6.1	Gene evolution trials and evaluation of mutant *THI4s*
